# Annotations

**Published:** 1889-04-13

**Authors:** 


### Annotations.

**"New "Tight-lace" Diseases.**

The "Medical Annual," a scientific record of the medical progress of the year, devotes a good deal of space to those diseases of women which are generally believed to originate from tight lacing. "During the last few years," says the "Annual," "several affections which are found in women with much greater frequency than in men have been claimed by independent writers in different parts of the world as the result of compression. . . . The most important are anæmia, ulcer of the stomach, gall-stones, movable kidney." This is a formidable array, and it must be noted that it is not a catalogue of all the diseases said to be produced by tight lacing, but only a list of a few which have been recently added to a very much longer series. "Anæmia" itself, in its "pernicious" form, is, as a rule, rapidly and hopelessly fatal. But even when it is not "pernicious," it is the prolific parent of manifold diseases, some of which are fatal, whilst almost all are extremely disabling and distressing. "Ulcer of the stomach" has an alarming sound even to the "lay" ear; but the sound is not nearly so alarming as the reality. When a doctor is convinced that he has met with a case of undoubted ulcer of the stomach, he anticipates weeks or months of misery for the patient and of harassing care for himself. He knows that recovery is possible; but he knows also that in many instances the chances are largely in favour of death. Treatment in many cases is quite powerless. One day the patient may be walking about, filling the air with complaint of her troubles. The next she may be dead, with a small perforation in the wall of the stomach as the result of the ulcerative process. "Gall-stones" it is unnecessary to dwell upon. The pain and danger of these are known almost universally. "Movable kidney," though less immediately painful and dangerous, is a condition which no woman who wishes to be well should for a moment run the risk of becoming acquainted with. This is the barest summary of facts, which might be so set forth as to appear truly appalling. Will any woman reader be frightened into reason? The answer, unhappily, is not even doubtful. She will not. But in case such a phenomenon should occur, the following suggestions of the "Annual" are worthy of her consideration. "The one thing that is most objectionable is the formation of an artificial waist. . . . . To simply order the removal of stays will be found altogether insufficient. . . . . for stays are undoubtedly a protection against the tight ligature of skirts which accompanies their use. The only satisfactory way is to *abolish both*. . . . . Every article of clothing, whether of upper or under garments, is to be made in combination, or without division at the waist. The weight of each garment is then borne mainly by the shoulders and bust, and no constriction of the waist is necessary." It is useless to appeal to the wearers of ladies' clothing, the case must be carried to the makers. Will Worth do anything? If he will not, will those educated and titled ladies who are said to have devoted their talents to the art of dressing their sisters, come to the rescue? There is a splendid field for enterprise and originality of mind.

**Skim Milk as a Food.**

Nothing more clearly shows the injury done to the whole population of the country by the separation of the people from the soil than the complete ignorance which almost universally prevails in towns as to the value of certain foodstuffs. A discussion has been carried on for some time in the columns of a morning contemporary about "skim milk." A correspondent, who professed to know the trade, boldly affirmed that skim milk was of little or no value as a foodstuff for human beings. If he had made such a statement at many a village pump in an evening after work hours, a score of farm labourers and their rosy-faced sons would have laughed him to scorn. Chemists give the results of their analyses, and these are entirely in favour of the popular rural opinion, that skim milk, with plenty of bread, constitutes an almost sufficient diet in itself for boys and girls. The popular mind does not readily pin its faith to chemical analyses, but in the present case the chemists, in seeking for proof, have performed a work of supererogation. The "proof of the pudding is in the eating," and of the skim milk in the drinking. The present writer has had a long familiarity with agriculture in almost all its branches, in addition to his medical training and experience. He has seen over and over again pigs and calves reared entirely on skim milk, or as nearly so as possible. These animals, although they will not become so fat on skimmed as on whole milk, will yet grow and thrive remarkably well. Similarly, he has seen the boys and girls of labourers, whose parents could have unlimited supplies of skim milk from a neighbouring farm, and other boys and girls whose parents were deprived of any such supply. The contrast between the children who had abundance of skim milk with their bread and potatoes, and those who had only water or "sloppy" tea, was most marked and unquestionable. In the one case, the milk seemed to be all that was required as an addition to bread and potatoes, with occasionally a little meat, to produce perfect health. Even the men in the Northern farm-houses, who feed in their master's kitchen, and at his cost, and whose supplies of beef, mutton, and bacon are practically unlimited, would feel it a great deprivation to be robbed of their skim milk. It does not seem to be generally known, or indeed known at all, that a little suet, cut fine, and boiled well in skim milk makes a most palatable food, and gives to the skim milk all the fat it requires. It would be an incalculable advantage to the poor of large towns if each child could have at least a quart of sweet skimmed milk every day, either with or without the added suet boiled into it. The man who, by any means, deprives the mass of the people of the use of a cheap and abundant article of food does more harm than a pestilence or a despotic ruler.

**Hath Music Charms?**

"Music hath charms to soothe the savage breast." So sang the poet, and so all men have believed. But however truly the poet may have spoken in regard to "savage" breasts, it appears quite certain that in "civilised" bosoms the effect of music may be the very opposite of "soothing." The North of England, and particularly the manufacturing districts of West Yorkshire and Lancashire, delight in "contests" of all sorts. "Dog-running" is popular there, and "pigeon flying," and "cockfighting," and, it is to be feared, "pugilism." But the love of fighting shows itself in milder forms, and "contests" of rival brass bands are among the chief pastimes of the summer season. Music, therefore, in these cases is put to quite a different use from that of "soothing"; it is, in fact, merely an additional method of defying and conquering your enemy. Unhappily, every brass band cannot carry off the "blue riband" of victory. Only one can take first prize; many must always go home without any prize at all. That the North countryman refuses to understand. Like our splendid Peninsular veterans, he never knows when he is beaten. This dogged determination of his to be on the winning side is likely to cost him dear in the immediate future. Mr. Charles Godfrey, so long and so well known as an adjudicator in band contests, declines, it is stated, to act as a "judge" any more, either in Lancashire or Yorkshire, owing to some rough usage he received at Bellevue last year at the hands of disappointed competitors. North country people will not be surprised to hear of the rough usage, however deeply they may regret it. They know the temper of their fellow countrymen all too well. Not very long ago, in another part of the country, it is said to have been quite a common thing for those bands to whom no prize had been awarded to lay prompt hands on the adjudicators and "duck" them in the nearest pond. Of course all this has its amusing side. But membership in a brass band is often of great educational and health value to thousands of men who are cooped up in factories and coalpits five or six days a week, and it is eminently desirable that all possible stimulus shall be given to the men to practise and excel in their work. No stimulus is stronger than that of competition, and no competition is worth anything except that which is adjudicated upon by competent judges. To adopt a course of conduct which drives the best judges from the judgment-seat is a death-blow to all excellence. In the interests of a healthy and educational recreation of great value, it may be hoped that Mr. Charles Godfrey will reconsider his decision, and that Lancashire and Yorkshire men will learn to accept defeat with a better grace.

